# Study on the Performance and Mechanism of Cold-Recycled Asphalt Based on Permeable Recycling Agent

**DOI:** 10.3390/ma16196464

**Published:** 2023-09-28

**Authors:** Peifeng Cheng, Pengcheng Qiao, Chunmeng Zheng, Ziyu Liu, Zhanming Zhang, Yiming Li

**Affiliations:** 1 School of Civil and Transportation Engineering, Northeast Forestry University (NEFU), Harbin 150040, China; chengpeifeng@nefu.edu.cn (P.C.); qpc_123@nefu.edu.cn (P.Q.); zhengchunmeng123@nefu.edu.cn (C.Z.); lzy2022@nefu.edu.cn (Z.L.); 2 Jiangsu Expressway Engineering Maintenance Technology Co., Ltd., Nanjing 211106, China; zhanming974@163.com

**Keywords:** aged asphalt, cold-recycled asphalt, permeable recycling agent

## Abstract

In order to investigate the influence of recycling agent composition on the recycling effect of aged asphalt in the cold recycling process, the design and optimization of cold recycling agent composition were performed through the central composite design-response surface method combined with the dynamic shear rheometer (DSR) test and the bending beam rheometer (BBR) test. The molecular weight distribution and component changes in aged asphalt before and after the addition of a cold recycling agent were also analyzed by gel permeation chromatography (GPC) and hydrogen-flame ionization test. The results showed that the permeable cold recycling agent has a recycling effect on the aged asphalt, but its effectiveness is greatly affected by recycling agent composition. The best recycling effect was achieved when the ratio of aromatic oil and penetrant in the cold recycling agent was 61.2:38.8, respectively. In terms of the recycling agent and aromatic functional groups in the aromatic oil, the aromatics in the recycling agent are derived from the aromatic oils, and the penetrant is only fused and permeated with the aromatic oils. After the admixture of the cold recycling agent, the penetrant in the recycling agent allows the aromatic oil to enter the aged asphalt at room temperature. The light components volatilized by aging are replenished, allowing the aged asphalt to recover some of its properties.

## 1. Introduction

With more emphasis on the concept of sustainable development of road engineering, pavement materials [1,2,3] have gradually become a research hotspot. Currently, the commonly used asphalt pavement recycling technology can be divided into hot recycling and cold recycling according to the construction temperature. Although hot recycling is more effective in improving the road performance of reclaimed asphalt pavement (RAP) than cold recycling, the higher construction temperature also affects the environment with increased amounts of harmful gases and carbon emissions. Due to the relatively low construction temperature, cold recycling can effectively decrease the harmful gas and heat emissions generated by RAP during the paving process and reduce energy consumption. However, in the process of cold recycling, RAP is often regarded as a “black stone” [4]. As a result, the old asphalt on the surface of RAP cannot be fully utilized, decreasing the utilization rate of the old asphalt on the surface of RAP and increasing the usage of new asphalt. Mao [5] argued that the surface of RAP is covered with old asphalt. If its performance can be restored, its utilization in the recycling process can be improved. According to Ashimova et al. [6], a recycling agent can effectively restore the properties of old asphalt on the surface of RAP, making it useful in recycled asphalt mixtures. Abraham et al. [7] believed that the light components of the recycling agent could be added to regenerate the old asphalt. All these findings indicate that research on recycling agents is necessary for the recycling of old asphalt. Currently, recycling agents [8,9,10] are mostly used in hot recycling. Due to the temperature limitation of cold recycling, the oil and lightweight components required for recycling cannot penetrate and replenish the old asphalt at room temperature. Therefore, the development of cold recycling agents is of great significance.

Asphalt consists of four kinds of components [11], including saturates, aromatics, resins, and asphaltenes. Xiao et al. [12] concluded that the aging of asphalt is mainly due to the decrease in light components (such as saturates and aromatics) and the increase in heavy components (such as colloids and asphaltenes) after high temperatures and oxidation [13,14,15]. As a result, the high-temperature performance of asphalt rises, and the low-temperature one decreases. The main components of the recycling agent for cold-recycled asphalt are aromatic oils [16] and penetrants. Aromatic oils can replenish saturates and aromatics in aged asphalt [17], i.e., as well as the light components [18]. The main component of the penetrant is methylene chloride, which is an organic solvent that can penetrate the old asphalt to restore some of its properties when fused with aromatic oils. Recycling agents are generally used in hot recycling to replenish the components and enhance the low-temperature crack resistance of the old asphalt [19,20,21]. Nevertheless, these agents are rarely used in cold recycling.

**Figure 1:**
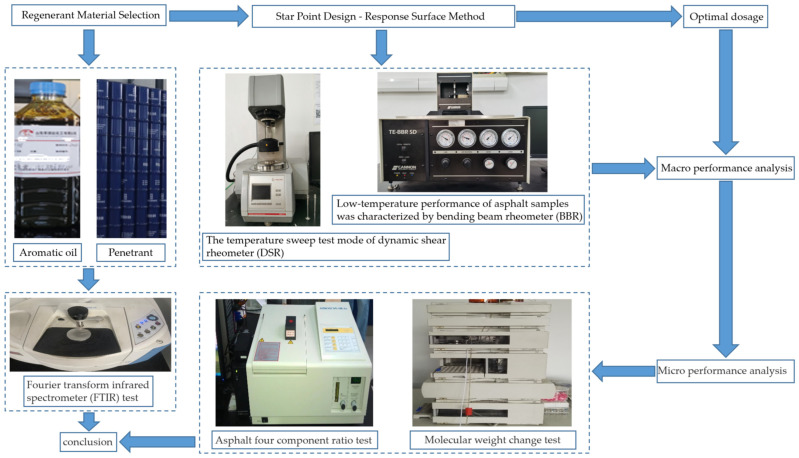
Technology diagram.

Based on the discussion above, the recycling agent for cold-recycled asphalt is proposed in this study. It can penetrate the old asphalt on the surface of RAP at room temperature and activate the old asphalt, which is effective in the synthesis process of cold-recycled asphalt, thus achieving the purpose of resource-saving. The optimal ratio for preparing recycling agents for cold-recycled asphalts is investigated using the central composite design-response surface method. The proportional relationship among recycling agent components is obtained by analyzing the high- and low-temperature performance and fatigue life of aged asphalt after adding the recycling agent, as well as by comparing the functional groups of the recycling agent with those of aromatic oils. Furthermore, the recycling mechanism of the recycling agent is analyzed according to molecular weight and component changes, and the technical route is shown in Figure 1.

## 2. Materials and Methods

This section introduces the experimental materials, optimal mixing ratio for preparing cold recycled permeable regenerative agents, and experimental methods for verifying their regenerative performance, as well as the preparation method of cold recycled asphalt.

### 2.1. Test Material

The category of neat asphalt in the test is PG 64-22, and the aged asphalt was prepared by heating the neat asphalt through a rotary film oven [22] at 163 °C for 5 h. The main technical indicators of neat asphalt and aged asphalt are shown in Table 1.

The cold recycling agent used in the test was produced by blending aromatic oils and penetrants in a certain proportion. Aromatic oils are bought directly, and their technical specifications are shown in Table 2. The penetrant is a composite penetrant with methylene chloride as the main ingredient, and its technical specifications are shown in Table 3.

### 2.2. Test Method

This section introduces the methods for preparing the optimal mixing ratio of regenerant components and verifying their regeneration performance in this study.

#### 2.2.1. Central Composite Design-Response Surface Method

In this study, a 2-factor, 5-level experiment was carried out to optimize the composition of the cold recycling agent using the central composite design-response surface method [23,24]. The factor code level and experimental design are shown in Table 4 and Table 5, respectively. In order to optimize the composition of cold recycling agents for the best overall performance, the recycling effect was evaluated by calculating the OD value according to Equations (1) and (2). The OD value means the abbreviation of “overall desirability” [25]. In the case that many indicators exist in the experimental results, the optimal conditions for each indicator may be contradictory. The OD value was used to integrate all indicators into a single value and reflect the overall results.

The changes in asphalt performance indexes before and after recycling were introduced as the evaluation basis to analyze the effect of recycling agents on aged asphalts. The smaller difference between recycled asphalt and neat asphalt indicates closer properties and a better recycling effect. The difference in fatigue life between aged asphalt and neat asphalt after adding the recycling agent is taken as an absolute value, and the test design and results are further analyzed.

(1) $$d_{\max}=(y_{i}-y_{\min})/(y_{\max}-y_{\min})$$

(2) $$d_{\min}=(y_{\max}-y_{i})/(y_{\max}-y_{\min})$$

where y is the value of the indicator, i is the test number, $d_{\max}$ denotes the factor for which the larger value is better, $d_{\min}$ represents the factor for which the smaller value is better, $y_{\max}$ is the maximum value of the indicator in each column, and $y_{\min}$ is the minimum value of the indicator in each column. The geometric mean of each indicator after normalization was calculated through Equation (3) to obtain the overall normalized value:

(3) $$OD={\left(d_{1}d_{2}d_{3}\cdot \cdot \cdot d_{n}\right)}^{1/n}$$

where n is the number of indicators and d is the normalized value.

#### 2.2.2. Specimen Preparation for Dynamic Shear Rheometer (DSR) Test and Bending Beam Rheometer (BBR) Test

**Figure 2:**
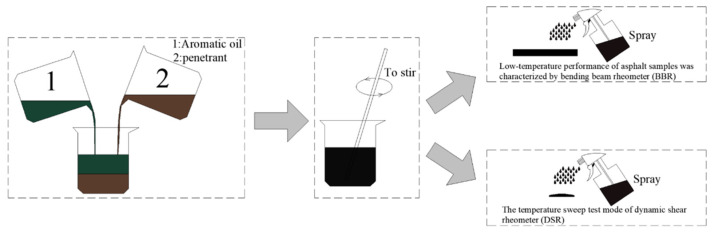
The preparation method of the cold recycled asphalt regenerant sample is shown in the figure.

The asphalt aged for 5 h was poured into the molds for the DSR and BBR tests. The demolding was performed after cooling, and the different dosages of recycling agents were evenly sprayed onto the specimen surface, as shown in Figure 2. After 20 min of static infiltration, the excess recycling agent was wiped off the surface, and the specimen was subjected to the DSR test or placed in a water bath at −12 °C for 1 h. Then, the BBR test was performed with the recycling agent, whose dosage was 8% of the mass of the asphalt specimen.

#### 2.2.3. Temperature Sweep Test

In order to investigate the effect of recycling agent addition on the high-temperature performance of aged asphalt, the high-temperature performance of neat asphalt, aged asphalt, and aged asphalt with different dosages of recycling agent was analyzed through the temperature sweep test. Moreover, the high-temperature performance and fatigue performance of asphalt were evaluated using phase angle (δ), complex shear modulus $\left(G^{*}\right)$ [26], storage modulus $\left(\left[G\prime \left(\omega \right)\right]\right)$, rutting factor $\left(\left|G^{*}\right|\sin\delta \right)$ [27], and fatigue factor $\left(G^{*}/\sin\delta \right)$. The load frequency of the test was 10 rad/s, the test temperature was 52~76 °C (6 °C interval), the diameter of the parallel plate was 25 mm, and the gap of the parallel plate was (2 ± 0.05) mm.

#### 2.2.4. Linear Amplitude Sweep (LAS) Test

The fatigue performance of asphalt is the main indicator to evaluate the durability performance of asphalt. In order to ensure the excellent durability of recycled asphalt, its fatigue properties need to be analyzed.

Currently, the commonly used methods for evaluating the fatigue performance of asphalt admixtures include time sweep, LAS, and fatigue factors. Among these methods, the LAS is a new type of test to evaluate the fatigue performance of asphalt based on the theory of viscoelastic continuum damage.

Through the LAS, the development of continuum damage in asphalt under repetitive loading can be analyzed. The VECD model constructed with the obtained fatigue equations can effectively predict and evaluate the fatigue performance of asphalt [28,29,30]. The specific test procedure for LAS is divided into two parts: frequency sweep and amplitude sweep. First, the damage analysis parameter α was obtained by performing a frequency sweep on the asphalt within a strain level of 0.1% and a frequency of 0.2 to 30 Hz. Then, the amplitude sweep was performed at a loading frequency of 10 Hz with a linear increase in loading amplitude from 0.1% to 30%. The diameter of the parallel plate was 8 mm, and the distance between the parallel plates was taken as (2 ± 0.05) mm.

#### 2.2.5. BBR Test

The flexural creep modulus *S* and creep rate *m* can be obtained from the BBR test of asphalt, and these two indicators are used to evaluate the low-temperature cracking resistance of the aged asphalt after the addition of recycling agents. In general, a larger *S* value indicates that the asphalt is harder and more prone to cracking at low temperatures; a larger *m* value indicates the better stress relaxation of the asphalt (i.e., the slower accumulation of stress leads to better low-temperature performance) [31,32]. Therefore, recycled asphalt has better flexibility and relaxation ability with a smaller *S* and a larger *m*. In addition, the low-temperature coefficient (k=S/m) [33] was introduced to comprehensively evaluate the low-temperature performance of aged asphalt after adding the recycling agent.

Before the start of each test, the instrument was calibrated using a stainless steel beam with a thickness of 1.0–1.6 mm to ensure that the water bath temperature reached the test temperature. After maintaining the test temperature for 65 min, the specimen was placed on the support, and the temperature of the thermostatic bath was controlled within ±0.1 °C of the experimental temperature. A load contact of 40 mN was manually applied to the specimen, and the time of load application was less than 10 s to ensure that the specimen and the load tip were in contact with each other.

#### 2.2.6. Fourier Transform Infrared Spectrometer (FTIR) Tests

Attenuated total reflection [34] was conducted to analyze the chemical structure of the aromatic oils and recycling agents using a Nicolet iS50 FTIR from Thermo Fisher (Waltham, MA, USA). The functional groups of the light components in the aromatic oils and recycling agent and the performance of the recycling agents on aged asphalt were determined. Aromatic oil and recycling agent samples were tested separately in a specimen cell. Asphalt samples can be scanned directly without pre-processing, and the reflective signals from the surface can be captured to analyze the organic components on the surface, as well as the structural information of the inorganic materials. Before the test, it is necessary to pre-heat the infrared light meter for 30 min. Then, the background scanning was performed under the same conditions, with a resolution of 4 cm^−1^, a scan number of 32 times, and a scanning range of 4000~500 cm^−1^. After collecting IR spectral data for each sample, the surface of the crystal plate was wiped and cleaned using a carbon disulfide solution. Two parallel specimens were prepared for each group, which were required to be scanned twice to ensure repeatability and overcome errors due to unstable measurement environments and uneven aging of the asphalt.

#### 2.2.7. Gel Chromatography Test

Gel permeation chromatography (GPC) is a type of liquid chromatography that can be used to determine the molecular weight and molecular weight distribution in asphalt for the investigation of its aging properties. The indicators used to evaluate the molecular weight distribution are the weight-average molecular weight $\left(M_{w}\right)$ and the number-average molecular weight $\left(M_{n}\right)$, of which $M_{n}$ is often used to reflect the trend of small molecular weights while $M_{w}$ can reflect the change in large molecular weights, as expressed by Equations (4) and (5), respectively [35,36,37]. The model of the test apparatus was Agilent 1100 with a refractive index detector. The test temperature was 30 °C, the eluent was tetrahydrofuran, the flow rate of the eluent was 1.0 mL/min, the concentration of the specimen was 1.0 g/L, and the injection volume of each test was 0.05 mL.

(4) $$M_{n}=\sum N_{i}*M_{i}/\sum N_{i}=\sum W_{i}/\sum W_{i}*M_{i-1}$$

(5) $$M_{w}=\sum W_{i}*M_{i}/\sum W_{i}$$

#### 2.2.8. Thin-Layer Chromatography-Flame Ionization Detection (TLC-FID) Test

Compared with the four-component test for standard asphalt, TLC-FID has the characteristics of less sample dosage, high precision, good repeatability, short test time, and less pollution [38,39]. This test can reflect the changes in each component in the aged asphalt after adding the recycling agent. The recycling effect of aged asphalt can be observed when compared to the neat asphalt composition.

Approximately 50 μg of the sample was extracted through the sample syringe and dispensed at 2 cm from the top of the rod. The solvent was removed by evaporation at room temperature and placed in an unfolding bath filled with n-heptane for unfolding. When the solvent n-heptane carrying saturates reached 11~12 cm of the rod, the rod was removed and dried for 10 min and then put into the unfolding tank containing toluene. When the toluene carrying the aromatic component is moved to 8 cm or 9 cm, the rod is taken out and dried for 10 min and then put into the unfolding tank with ethanol and toluene. Finally, when the solvents of ethanol and toluene brought resins to 4~5 cm, the specimen was taken out and placed in an 80 °C constant temperature box to dry for 10 min.

## 3. Results and Analysis

### 3.1. Test Results of Central Composite Design for Recycling Agents

The results of the central composite design-response surface method test and the fitted values are shown in Table 6. The nonlinear regression analysis was performed using the Design Expert 11 software (https://www.statease.com/software/design-expert/), and Equation (6) was obtained after fitting (multi-correlation coefficient $R^{2}=0.9184$):

(6) $$OD=0.7925-0.0474x_{1}+0.2016x_{2}+0.1470x_{1}x_{2}-0.0716x_{1}^{2}-0.2330x_{2}^{2}$$

where $x_{1}$ is aromatic oil content and $x_{2}$ is penetrant content.

**Figure 3:**
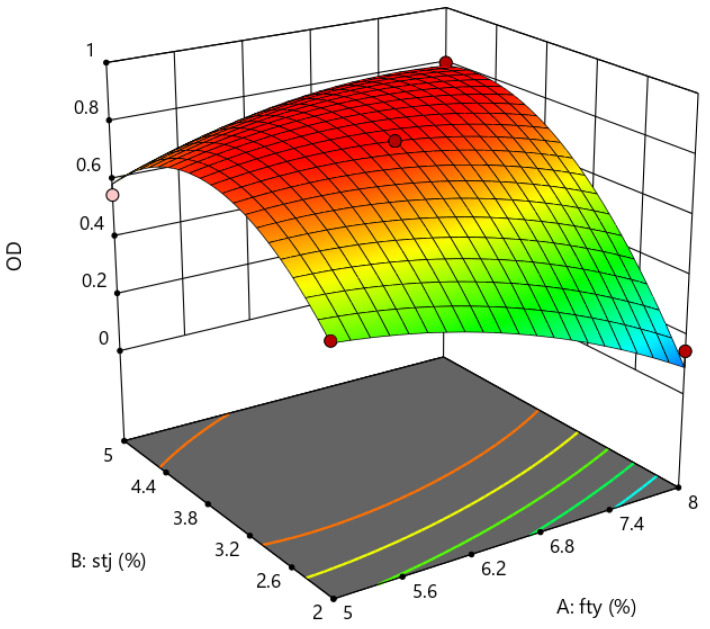
The effect surface of the ratio (code) of penetrant and aromatic oil to regenerant on OD value.

*OD* is the response surface depicted by one dependent variable and two independent variables, and the three-dimensional plot is shown in Figure 3.

It can be seen from Figure 3 that the composition of the recycling agent has a greater impact on the OD value. The figure shows a trend that the OD value increases first and then decreases with the increase in penetrant content in the recycling agent. In contrast, the OD value first increases and then decreases with the decreasing content of aromatic oils. This result indicates that the two components of the cold recycling agent can affect the recycling performance and is reflected by the OD value. The highest OD value was achieved when the proportion of aromatic oil and penetrant recycling agent was 61.2% and 38.8% in the recycling agent, respectively, indicating the best recycling effect. The optimal ratio of the recycling agent was experimentally verified, and the OD value obtained from the model was 0.839. According to the optimal dosage of the two components (i.e., 61.2% aromatic oil and 38.8% penetrant), the recycling agent was produced and added to the aged asphalt, with a deviation of 3.2% (Table 7). The small deviation of the predicted value from the actual value indicates that the developed mathematical model can predict the optimal dosages of the two components in the recycling agent for the cold-recycled asphalt.

### 3.2. Results Analysis of the Infrared Spectroscopy Test

**Figure 4:**
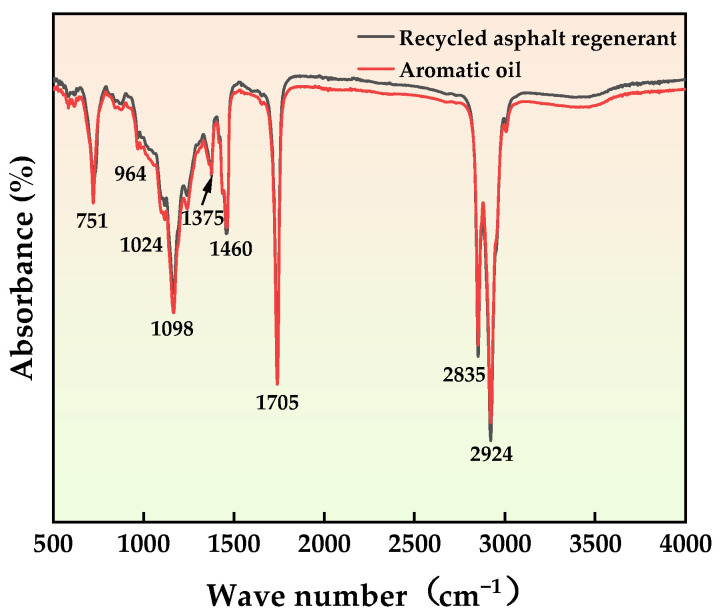
Infrared spectra of aromatic oil and regenerant.

The infrared spectra of the aromatic oil and the cold recycling agent were scanned, as shown in Figure 4. The results show strong vibration peaks of aromatic C-H and C=C stretching at 2924 cm^−1^, 2835 cm^−1^, and 1705 cm^−1^, indicating that the aromatic oil is rich in aromatic light components. The recycling agents are generated after mixing aromatic oils with penetrants. The infrared spectra of the recycling agent and the aromatic oil are basically the same, reflecting that the substances in the recycling agent that can replenish the light component of the aged asphalt come from the aromatic oil. It also demonstrates that the penetrant in the recycling agent fused with the aromatic oil only serves to penetrate the aged asphalt.

### 3.3. Results Analysis of the Temperature Sweep Test

**Figure 5:**
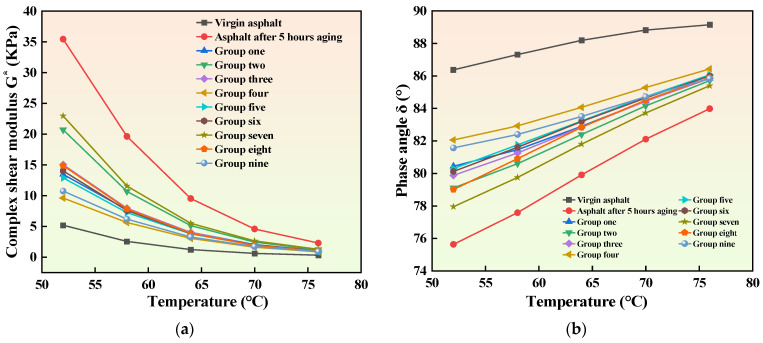
Temperature scanning test results. (**a**) Complex shear modulus and time relationship diagram. (**b**) The phase angle and time diagram.

The results of the temperature sweep test are shown in Figure 5. As the temperature increases, the composite shear modulus of asphalt gradually decreases while the phase angle gradually increases. Compared to unaged asphalt, the aged asphalt shows a significant increase in complex shear modulus and a decrease in phase angle after 5 h of aging. This result indicates that aging changes the viscoelastic ratio of asphalt, and aged asphalt has an increased elastic component and hardness. The addition of a recycling agent has a certain softening effect on the aged asphalt. After adding the recycling agent, the asphalt complex shear modulus decreases, and the phase angle increases, but this effect is strongly influenced by the composition of the recycling agent. The high-temperature performance of the asphalt specimens in Group IV is most similar to that of the unaged neat asphalt, suggesting that the recycling agent in Group IV has the optimal effect compared to other recycling agents. The superior recycling performance is due to different ratios of penetrant and aromatic oil in the recycling agent. A reasonable ratio of penetrant and aromatic oil contributes to the more complete penetration of aromatic oil into aging asphalt at room temperature, thus replenishing the light components volatilized from the asphalt due to aging. On the other hand, excessive penetrant means less aromatic oil in the recycling agent, which cannot effectively replenish the light components volatilized from the asphalt, leading to a poorer recycling effect and a higher modulus.

### 3.4. Results Analysis of the Temperature Sweep Test

**Figure 6:**
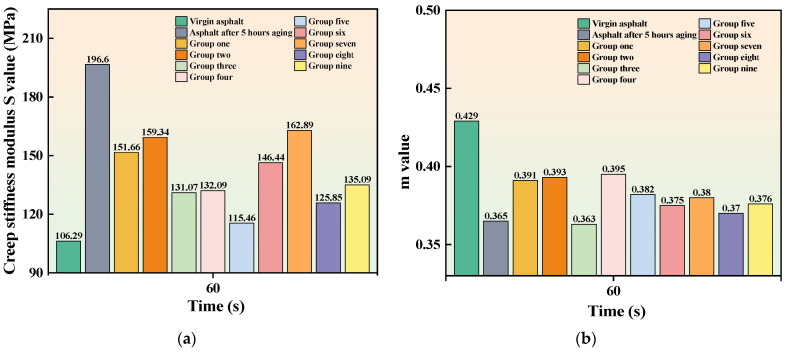
Bending beam rheometer test results (**a**) Creep stiffness modulus S value and time diagram (**b**) m value and time diagram.

**Figure 7:**
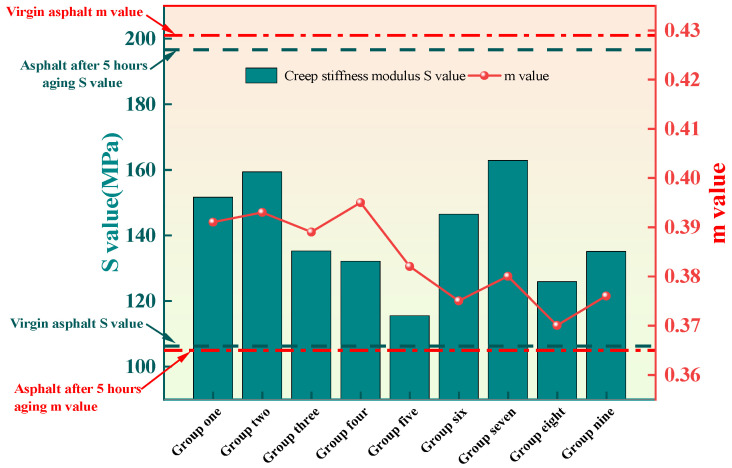
S value and m value.

**Figure 8:**
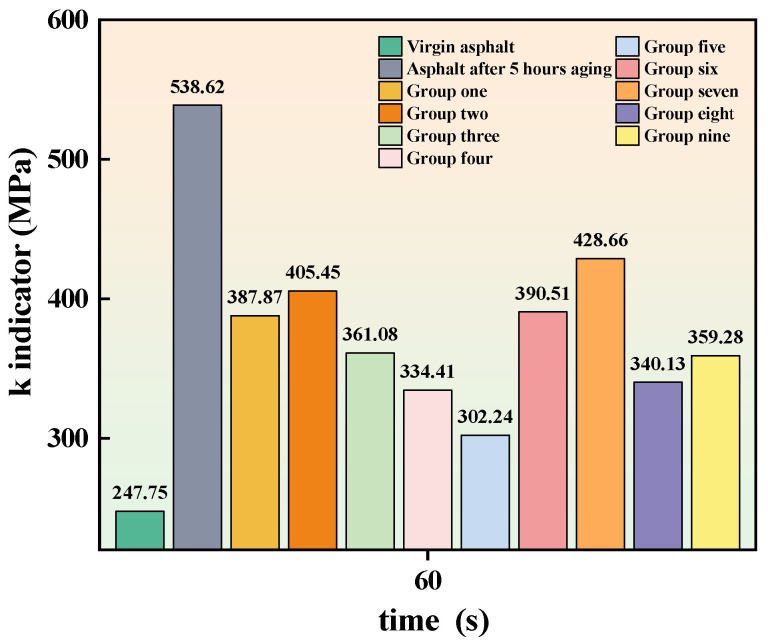
k indicator.

The results of the BBR test are shown in Figure 6 and Figure 7. It can be seen that the S value of neat asphalt increases after aging while the m value decreases, indicating a decrease in the cracking resistance and the low-temperature performance of the asphalt. After adding the cold recycling agent, the S value of aged asphalt decreases, and the m value increases, suggesting that the addition of the recycling agent can improve the low-temperature performance of aged asphalt. The previous study argued that the low-temperature performance of asphalt cannot be comprehensively evaluated by S value or m value alone [40]. Therefore, the low-temperature coefficient k, which is obtained by calculating the ratio of S to m, is a better way to evaluate the low-temperature performance of asphalt, and the calculation results are shown in Figure 8. It can be seen that the k of asphalt specimens in Group V is closest to that of the unaged asphalt, indicating that this group of specimens has the best low-temperature performance after recycling compared to the other groups. A reasonable mixing ratio of the recycling agent makes the aromatic oil and penetrant fully integrated. As a result, penetrant with lightweight components can penetrate the aged asphalt, enabling the recycling of the aged asphalt. When the ratio of penetrant in the recycling agent is low, the penetrant cannot mix with the excess aromatic oils and cannot fully penetrate the old asphalt. On the contrary, although more penetrant can be fully integrated with the light components in the aromatic oil, insufficient aromatic oil cannot replenish the aging asphalt with enough light components to achieve a good recycling effect.

### 3.5. Result Analysis of the LAS Test

**Figure 9:**
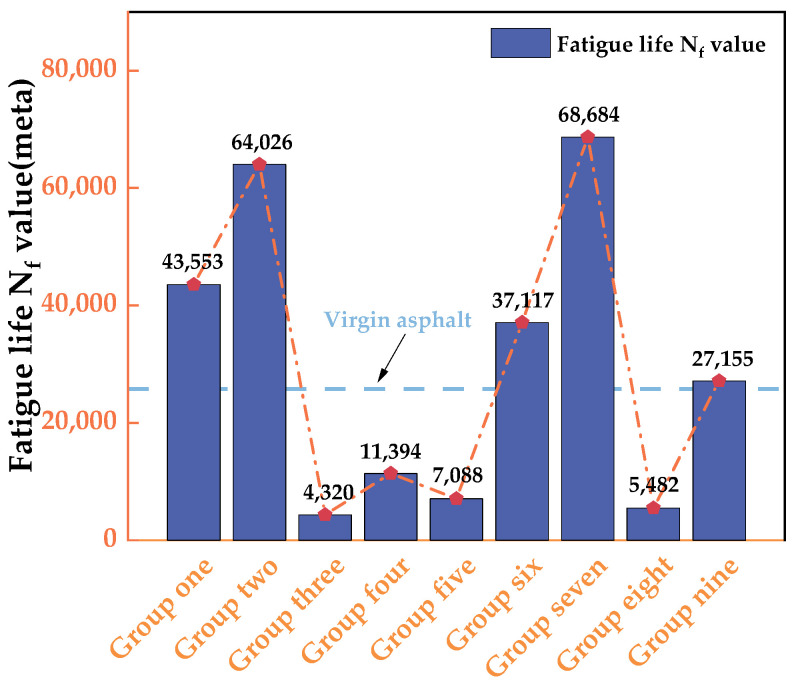
The fatigue life value of each group.

According to the results of the LAS test, the fatigue life value (N_f_) of the fitted asphalt is obtained, as shown in Figure 9. It can be seen that after 5 h of aging, the N_f_ of neat asphalt increases from 25,761 cycles to 143,772 cycles. The fatigue life of aged asphalt decreases after the addition of the recycling agent, indicating that the addition of the recycling agent has a recycling effect on aged asphalt. The fatigue life of aged asphalt after adding the recycling agent at the dosage of Group VII is 68,684 cycles, which is closest to that of the aged asphalt. After adding the recycling agent at the dosage of Group III into the aged asphalt, the fatigue life is 4320, and the recycling agent at a dosage of Group IX leads to a fatigue life of 27,155, which is the closest to that of the neat asphalt. The reason for the situation above is that the proportion of penetrants in the recycling agent of Group VII is low, resulting in incomplete penetration of oil into the aged asphalt and a poor recycling effect. The fatigue life decreases as the dosage of penetrant in the recycling agent increases. However, when the proportion of penetrant is too high, the fatigue life is lower than that of the neat asphalt. The main component of the penetrant is methylene chloride, which softens the aged asphalt when in contact with it. An excess dosage of penetrant in the recycling agent increases the softening degree of the asphalt, leading to a decreased fatigue life.

### 3.6. Result Analysis of the GPC Test

**Figure 10:**
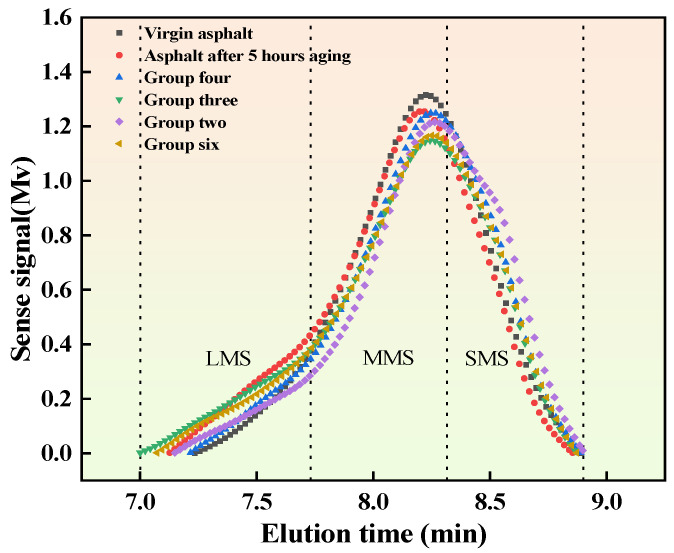
GPC spectra of asphalt molecular weight.

Typically, the macroscopic properties of asphalt can be reflected by its microscopic molecular structure as well as its molecular weight distribution. The recycling agents in the dosage of Group II, Group III, Group IV, and Group VI were selected and added to the aged asphalt, and the corresponding samples were subjected to the GPC test. The results of the test are shown in Figure 10. The spectra are divided into large, medium, and small molecule regions, which are summarized in Table 8. It can be seen that the number of large molecules increases and the number of medium and small molecules decreases in aged asphalt compared to unaged asphalt. The addition of recycling agents replenishes small molecules in the aged asphalt, thus affecting the molecular weight distribution of the recycled asphalt. After adding the recycling agent, the large molecules in the asphalt decrease, and the small molecules increase, with the component ratio of asphalt being restored to a certain extent. The properties of asphalt are also recovered, but the recovery effect is significantly influenced by the ratio of aromatic oil to penetrant in the recycling agent. Based on the results of the GPC test, the high-temperature performance of the aged asphalt decreases after the addition of a recycling agent, and the low-temperature performance increases. In addition, the number of large molecules and small molecules in aged asphalt is close to that of neat asphalt, indicating that the recycling agent for cold-recycled asphalt can restore the performance of aged asphalt. According to the molecular weight distribution of aged asphalt with different dosages of recycling agents, the large molecule content of recycled asphalt with a dosage of Group II is 15.74%, which is higher than that of the other three groups. Its proportion of large molecules is also closer to that of aged asphalt, indicating that the dosage of Group II can lead to better high-temperature performance, further validating the best high-temperature performance brought by the dosage of Group II in the temperature sweep test. Compared with the other three groups, the dosage of Group III results in a higher proportion of small molecules (37.22%), indicating a better low-temperature performance of the asphalt than that in the other three groups. This result is consistent with the BBR test.

### 3.7. Result Analysis of the TLC-FID Test

**Figure 11:**
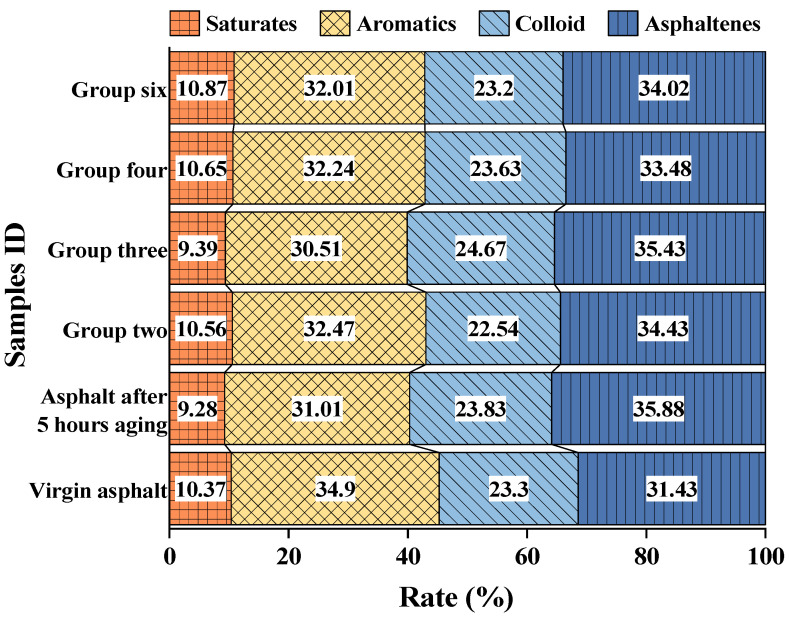
Asphalt four component quality analysis diagram.

As shown in Figure 11, the content of saturates and aromatics in neat asphalt decreases after aging, while the content of resins and asphaltenes increases. After adding different proportions of recycling agents to aged asphalt, the recovery degree of asphalt components varies. In order to evaluate the component replenishment after adding a recycling agent to aged asphalt, the colloidal stability index (*I_c_*) of asphalt [41] was introduced to assess the performance of the recycling agent. The structure of the asphalt colloidal structure is determined by the relative content of the components. The *I_c_* was calculated according to Equation (7). A smaller *I_c_* indicates that the colloidal structure is closer to the gel structure, and a larger *I_c_* indicates that the colloidal structure is closer to the soil structure. Similarly, systems with an *I_c_* close to that of the original asphalt are more stable. As shown in Table 9, R ‘ is the ratio of *I_c_* in each group to the *I_c_* of neat asphalt. After 5 h of aging, the I_c_ value of the asphalt was reduced to 87.21% of the neat asphalt. When the aged asphalt was incorporated with the recycling agent at the dosage of Group II, its *I_c_* value (1.223) was 87.81% of that of the neat asphalt, which is closest to the aged asphalt. After adding the recycling agent to the aged asphalt at the dosage of Group IV, its *I_c_* value (1.266) was 90.93% of that of the neat asphalt, which is closest to the neat asphalt with the best recycling effect.

The colloidal stability index *I_c_* can be calculated as follows:

(7) $${Ι}_{c}=\frac{R+A}{A_{SP}+S}$$

where *I_c_* is the colloidal stability index of asphalt, *R* is the mass fraction of resins in asphalt, %, *A* is the mass fraction of aromatics in asphalt, %, *ASP* is the mass fraction of asphaltenes in asphalt, %, and *S* is the mass fraction of saturates in asphalt, %.

## 4. Conclusions

A permeable cold recycled asphalt regeneration agent is synthesized and added to aged asphalt, and then the high- and low-temperature performance and fatigue life of aged asphalt after regeneration are analyzed using high- and low-temperature rheological performance and fatigue tests. The regeneration mechanism of permeable cold recycling agent on aged asphalt was explored through an infrared spectrum test, gel chromatography test, and component analysis test. The following conclusion is drawn:

(1) The permeable cold recycling agent has a certain recycling effect on the aged asphalt. As the proportion of penetrant in the recycling agent increases, the high-temperature performance of recycled asphalt tends to decrease and then increase, while its low-temperature performance tends to increase and then decrease.
(2) The recovery performance of recycled asphalt is strongly influenced by the mixing ratio of the cold recycling agent. The best recycling effect can be achieved when the ratio of aromatic oil and penetrant in the cold recycling agent is 61.2% and 38.8%, respectively.
(3) A cold recycling agent contained in the penetrant enables the aromatic oil to penetrate the aged asphalt at room temperature, replenishing the light components volatilized due to aging, improving the colloidal structure, and restoring the performance of the aged asphalt.
(4) From the aromatic functional groups of the cold recycling agent, it can be seen that all lightweight components are from the aromatic oil. The penetrant serves only as an organic solvent that blends with the aromatic oils and penetrates the asphalt.

This study introduces a synthesized permeable cold regeneration agent, analyzes and investigates its regeneration effect, and finds that it can achieve the effect of restoring certain performance of aged asphalt under room temperature conditions. However, the optimal permeation regeneration time and permeation depth mechanism of this regenerant are not yet clear. Further research could be conducted on the optimal permeation time and permeation mechanism of this regenerant, with the hope of being applied to practical engineering production.

## Figures and Tables

**Table 1:** Matrix asphalt and aging asphalt technical indicators.

Material Attributes	5 °C Ductility/cm	25 °C Penetration/(0.1 mm)	Softening Point/°C
The virgin asphalt	7.6	82.1	46.5
Asphalt after 5 h aging	—	51.7	54.0

**Table 2:** Aromatic oil technical index table.

Technical Index of Aromatic Oil	Unit	Test Results	Skills Requirement
Appearance	—	Brownish black	Brownish black
flash point	(°C)	230	≥180
Solidifying point	(°C)	10	≤20
Kinematic viscosity	(m^2^/s)	16.5	15–30
Density	(kg/m^3^)	0.96	0.90–1.05

**Table 3:** Penetrant technical index table.

Penetrant Properties	Unit	Test Results	Skills Requirement
1 mm thickness appearance	—	Brown	Black or brown
Solid content	(%)	24	≥45
Water permeability coefficient	(mL/min)	117	≤135
Viscosity	(Pa·s)	0.61	≥0.5
Friction mandrel value	(BPN)	47	≥32

**Table 4:** Factor code level and value.

Code Level	X1	X2
	(Aromatic Oil Content)	(Penetrant Content)
−1.414	4.4	1.4
−1	5	2
0	6.5	3.5
1	8	5
1.414	8.6	5.6

**Table 5:** Factor test design and component mixing scale.

Test Number	X1	X2	fty Design Value	stj Design Value	fty Actual Value	stj Actual Value
1	−1	−1	5	2	0.714	0.286
2	1	−1	8	2	0.8	0.2
3	−1	1	5	5	0.5	0.5
4	1	1	8	5	0.62	0.38
5	−1.414	0	4.4	3.5	0.56	0.44
6	1.414	0	8.6	3.5	0.711	0.289
7	0	−1.414	6.5	1.4	0.83	0.17
8	0	1.414	6.5	5.6	0.54	0.46
9	0	0	6.5	3.5	0.65	0.35
10	0	0	6.5	3.5	0.65	0.35
11	0	0	6.5	3.5	0.65	0.35
12	0	0	6.5	3.5	0.65	0.35
13	0	0	6.5	3.5	0.65	0.35

Note: fty is aromatic oil, stj is a penetrating agent.

**Table 6:** Experimental results.

Test Number	fty	stj	Rutting Factor (64 °C)/(kPa)	Rutting Factor (64 °C) Differentials/(kPa)	Creep Stiffness Modulus /(S value)	Creep Rate/ (m value)	k Indicator /(MPa)	k Indicator Differentials/(MPa)	N_f_/ (meta)	N_f_ Differentials/(meta)	OD
Virgin asphalt	—	—	1.19	0	106.29	0.429	247.75	0	25,761	0	—
Asphalt after 5 h aging	—	—	9.66	8.46	196.60	0.365	538.62	290.869	143,772	118,011	—
1	0.714	0.286	4.04	2.85	151.66	0.391	387.87	140.12	43,553	17,792	0.4884
2	0.8	0.2	5.13	3.94	159.34	0.393	405.45	157.69	64,026	38,265	0.1482
3	0.5	0.5	4.01	2.82	131.07	0.363	361.08	113.33	4320	21,441	0.5525
4	0.62	0.38	3.04	1.85	132.09	0.395	334.41	86.66	11,394	14,367	0.8003
5	0.56	0.44	3.79	2.60	115.46	0.382	302.24	54.49	7088	18,673	0.7413
6	0.711	0.289	3.83	2.64	146.44	0.375	390.51	142.76	37,117	11,355	0.5385
7	0.83	0.17	5.53	4.34	162.89	0.380	428.66	180.90	68,684	42,923	0.0000
8	0.54	0.46	3.87	2.68	125.85	0.370	340.13	92.38	5482	20,279	0.6340
9~13	0.65	0.35	3.27	2.08	135.09	0.376	359.28	111.53	27,155	1394	0.7925

Note: Group 9~Group 13 was a repeated test.

**Table 7:** Verification test results table.

Test number	fty	stj	Rutting Factor (64 °C)/ (kPa)	Rutting Factor (64 °C) Differentials/(kPa)	Creep Stiffness Modulus/ (S value)	Creep Rate (m value)	k Indicator/(MPa)	k Indicator Differentials/(MPa)	N_f_/ (meta)	N_f_ Differentials/ (meta)	OD	OD Value Deviation /(%)
1	0.612	0.388	2.86	1.67	135.21	0.389	347.594	99.843	16,915	8846	0.826	3.2

**Table 8:** Molecular weight calculation results.

Type	Molecular Dimension	Comparative Content (%)
	LMS	15.63
Virgin asphalt	MMS	57.32
	SMS	27.05
	LMS	16.67
Asphalt after 5 h aging	MMS	56.78
	SMS	26.55
	LMS	15.74
Group two	MMS	53.35
	SMS	30.91
	LMS	13.59
Group three	MMS	49.19
	SMS	37.22
	LMS	14.34
Group four	MMS	53.47
	SMS	32.19
	LMS	14.85
Group six	MMS	54.10
	SMS	31.04

Note: LMS is large molecules, MMS is middle molecules, and SMS is small molecules.

**Table 9:** Asphalt colloid index *I_c_* and R ‘ calculation results table.

Test Number	Virgin Asphalt	Asphalt after 5 h Aging	Group Two	Group Three	Group Four	Group Six
*I_C_*	1.392	1.214	1.223	1.231	1.266	1.228
R ‘(%)	100	87.21	87.81	88.42	90.93	88.17

## Data Availability

Not applicable.
